# Using Risk–Benefit Analysis and the Analytical Hierarchy Process to Decide on the Implementation of Rapid *Salmonella* Detection Methods in Large Poultry Industries

**DOI:** 10.1111/risa.70222

**Published:** 2026-03-24

**Authors:** Cris Rocha Pinto Magalhães, Nathanyelle Soraya Martins de Aquino, Eduardo Cesar Tondo

**Affiliations:** ^1^ Laboratório de Microbiologia e Controle de Alimentos, Instituto de Ciência e Tecnologia de Alimentos Universidade Federal do Rio Grande do Sul (ICTA/UFRGS) Porto Alegre Rio Grande do Sul Brazil

**Keywords:** chicken meat, food safety, multi‐criteria decision analysis (MCDA), rapid assay, risk management

## Abstract

Tools based on multi‐criteria decision analysis (MCDA) can be used in decision‐making processes related to food safety issues (FSI). We used risk–benefit analysis (RBA) and the analytic hierarchy process (AHP) to answer the following questions: What are the benefits and risks of using rapid *Salmonella* assays in large‐scale poultry industries? What are the benefits and risks of changing one implemented rapid method by another? In the RBA, we described the FSI, ranked *Salmonella* risk, and analyzed the benefits and costs of assays by considering seven criteria: supply capacity, international validation, cost of equipment, ease to perform, technical support, cost per sample, and time to obtain results. AHP, a mathematical framework based on pairwise comparisons, was used to confirm RBA findings. As a result, the RBA recommended the use of rapid assays in large poultry companies because they provide high benefits at low to high costs. However, the RBA was unable to identify the benefits of changing one implemented method by another because well‐known methods present similar characteristics, and the decision should be based on the best business proposals. The AHP analysis confirmed that faster assays bring higher benefits and could be companies’ choice, even costing more, but they have to attend to the main characteristics of well‐known assays, including equipment on loan. The main criteria for choosing rapid methods were prioritized as supply capacity, technical support, time to obtain results, and international validations. RBA and AHP reached equal conclusions; however, RBA was simpler to apply, whereas AHP enabled quantitative analysis.

## 1 Introduction

*Salmonella* is one of the most important food pathogens worldwide (Teklemariam et al. 2023; World Health Organization 2018) and its detection is important for improving the safety of poultry meat products. Therefore, the appropriate choice of *Salmonella* detection method is strategic for large‐scale poultry industries, because rapid and accurate results allow for faster safe food release and distribution.

Currently, there are several commercially available rapid *Salmonella* detection methods (Barrere et al. 2020; Sahu et al. 2019; Wang et al. 2015), and the choice of one is not an easy task, as their costs, benefits, and risks should be evaluated before implementation in companies’ laboratories. Some of the most important criteria to be considered when choosing a rapid *Salmonella* detection method are supply capacity, validation, cost, and time to obtain results that can vary from a few hours to a few days (Aquino et al. 2021; Nguyen et al. 2020). Even though it is assumed that a shorter time for results reduces the time to food release and distribution, reduces costs, and brings greater benefits, the change from a rapid method to a faster one may require additional investments, new documentation, and internal validations, increasing costs and risks for company management. For example, in large poultry companies, implementing a new method can take around a year. This investment of time and resources will become a favorable scenario in terms of cost–benefit when the rapid method reduces analysis time and its execution does not involve steps that are too different from those already being carried out in the company's laboratories.

Risk management is defined as coordinated activities to direct and control an organization regarding risk (International Organization for Standardization [ISO] 2018). In this context, risk is the effect of uncertainty on the objectives. If the aim of a rapid *Salmonella* detection method used in large‐scale poultry companies is to detect microorganisms as quickly as possible, the effect of choosing an inappropriate method can result in delays in results, possible commercial problems, and recalls. In the long term, all the tests carried out by the industry and inspection bodies are due to the risk *Salmonella* poses to public health.

Recently, the risk–benefit analysis (RBA) model was used to assist in food safety decision‐making by analyzing *Salmonella* control in Brazilian poultry meat (Tondo and Gonçalves 2021). RBA is a simple multi‐criteria decision analysis (MCDA) model that describes a specific food safety issue (FSI), ranks the risks associated with a particular pathogen, and identifies the urgency of the necessary control measures. Subsequently, the RBA analyzes the magnitude of the benefits and costs of adopting control measures, evaluating whether the benefits outweigh the risks or whether the costs are worth paying.

Another method used in risk management and decision‐making processes is the analytic hierarchy process (AHP), which is one of the main MCDA techniques (Andreolli et al. 2022; Chen et al. 2022; Dunn 2015; Hamidah et al. 2022; Ok et al. 2022; Radomska‐Zalas 2022; Saaty 1990). This method allows decision‐makers to rank the importance of various criteria in evaluating alternatives to address a specific issue (Saaty 1987) through pairwise comparisons using a nine‐point semantic scale (Saaty 2001) and by ensuring awareness of the consistency of their judgments (Saaty 1987).

As RBA can be helpful in the risk management of FSI (Tondo and Gonçalves 2021), we used the model to respond to two questions: (1) What are the benefits and risks of using rapid *Salmonella* detection assays in large‐scale poultry industries? (2) What are the benefits and risks of changing one implemented rapid method by another? We then performed an in‐depth analysis using the AHP methodology to evaluate the robustness of the RBA results and confirm the priorities of the most important criteria for choosing rapid *Salmonella* detection methods.

## 2 Materials and Methods

### 2.1 Risk–Benefit Analysis

**FIGURE 1:**
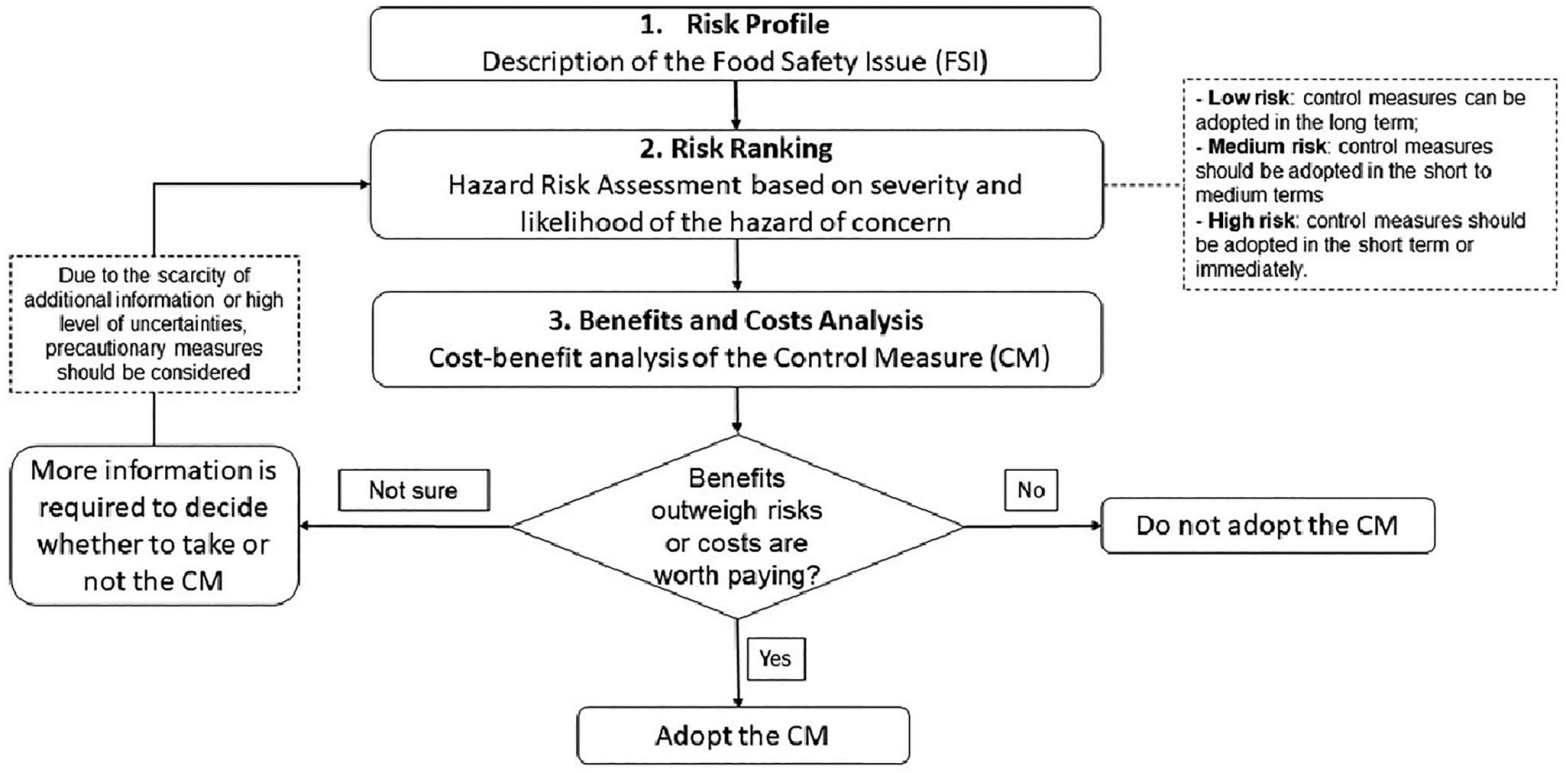
Framework of the risk–benefit analysis (RBA) model developed by the authors, inspired by Tondo and Gonçalves (2021).

The RBA model comprises three parts: (1) description of the FSI in the risk profile; (2) risk ranking for the hazard of concern; and (3) analysis of the magnitude of the benefits and costs involved in adopting control measures. Figure 1 illustrates the RBA model framework.

#### 2.1.1 Risk Profile

The risk profile presented in Table 4 consists of questions adapted from Annex 1 of the General Principles of Microbiological Risk Management (Codex Alimentarius Commission 2007; Tondo and Gonçalves 2021) to better describe the FSI of using rapid *Salmonella* detection methods in large poultry companies. The risk profile included the following questions:

1. What is the hazard being studied?
2. What is the description of the FSI?
3. How would you describe the hazard, severity of clinical manifestations, and its probability of being found in food?
4. What is the economic impact of using rapid methods?
5. What are the control measures for FSI?

#### 2.1.2 Risk Ranking

The risk associated with *Salmonella* was ranked according to the severity and likelihood categories described in the risk ranking matrix used in the RBA model (Tondo and Gonçalves 2021) (Table 5). In addition, assuming that the risk is the effect of uncertainty on the objectives (ISO 2018) and that the objective of a rapid *Salmonella* assay is the fast and accurate detection of this microorganism in foods, uncertainties are considered to be factors that can affect *Salmonella* detection, producing false‐positive or false‐negative results.

#### 2.1.3 Identification of Benefits and Costs

The RBA was conducted by four food safety experts, including a laboratory manager from a large poultry company, an experienced technical laboratory manager who provides services to the poultry industry, an experienced professor of food microbiology, and an experienced microbiologist responsible for testing and analyzing rapid *Salmonella* detection methods for research purposes. Following several technical meetings, the experts collectively listed seven key criteria as the most relevant for evaluating rapid *Salmonella* detection assays. They were supply capacity, international validation, cost of equipment, ease to perform, availability of technical support, cost per sample, and time to obtain results. However, among these priority criteria, experts were asked to rank the level of relevance between them, particularly when choosing a new rapid assay. Thus, the experts analyzed each of the seven identified criteria for rapid *Salmonella* detection assays using RBA, prioritizing them as essential, very important, important, moderately important, and irrelevant, according to their expert opinion (Table 6). As essential criteria, the experts considered those that, if not present, the rapid method would not be a possible choice for poultry companies.

The RBA focused on identifying the importance of the seven listed criteria for the three rapid *Salmonella* detection methods frequently used in laboratories in large Brazilian poultry industries. We assumed that the reality found in large‐scale Brazilian companies was similar.

We included a fourth method capable of detecting *Salmonella* in a shorter time (Aquino et al. 2021; Nguyen et al. 2020) to investigate whether the reduced analysis time of a method would justify it being selected over other well‐known assays. However, because this method is not yet commercially available, information on supply capacity and technical support availability is not provided (Table 6). In order to assess whether a company would pay more for a faster test, we hypothesized a 20% increase on the price of the most expensive method evaluated in this study. We assumed that a 20% increase in the price of rapid *Salmonella* detection assays would be significant based on the experts’ conversations with laboratory managers at large poultry companies, who frequently purchased rapid assays. The higher cost was justified by the more advanced technology of the new method. The cost of equipment of the fourth method was also considered higher than that of the others because performing the assay requires a luminometer, and this equipment would not be delivered on loan.

We considered the choice of the most appropriate rapid *Salmonella* detection method as the best control measure for FSI. The magnitudes of the benefits and costs associated with the adoption of the rapid method and possible changes in the rapid detection method were classified using the benefit and cost matrix (Table 1) of the RBA model (Tondo and Gonçalves 2021). The matrix was used considering the seven criteria of the rapid methods and the storage cost of one loaded truck (24 or 30 t) for frozen poultry meat waiting for delivery.

**TABLE 1:** Matrix for classifying the benefits and costs related to the adoption of measures to control food safety issues.

	Cost				
Benefit	Low	Medium	High	Very high	Excessive
Very high	A	A	A	B	C
High	A	A	A	B	C
Medium	A	A	A	B	C
Low	B	B	B	B	C
No benefit	C	C	C	C	C

Zone	Adoption of measure
Zone A	Control measure should be adopted
Zone B	Uncertainty zone, more information is required to decide whether to take or not the control measure. Due to the scarcity of additional information or a high level of uncertainty, precautionary measures should be considered
Zone C	Control measures should not be adopted

*Source*: Tondo and Gonçalves (2021).

The RBA methodology provides a simplified tool for food safety managers to assess the control measures. Tondo and Gonçalves (2021) developed a 5 × 5 matrix that classifies benefits and costs without precise quantification. The model uses categorical scales ranging from no benefit, low benefit, medium benefit, high benefit, and very high benefit. Costs are low, medium, high, very high, and exceptionally high. This approach acknowledges that control measures may have negligible benefits while recognizing that implementation always involves some investment. The matrix enables managers to conduct a flexible cost–benefit analysis, allowing the depth of investigation to be proportional to their specific decision‐making needs.

### 2.2 Analytic Hierarchy Process

When performing RBA, the model requested more information to determine whether there were benefits of changing one rapid method by another. This information was requested because the RBA was unable to classify the magnitude of the benefits of switching from one rapid method to another. This is because the characteristics of the evaluated methods were similar, except for the fourth method.

AHP was then applied to obtain more information about the prioritization of the seven criteria used to analyze the benefits and costs of rapid *Salmonella* detection methods, as well as to evaluate the robustness of the RBA results.

The AHP is a decision‐making model composed of three parts: (1) identifying and organizing decision objectives, criteria, and alternatives into a hierarchy; (2) evaluating pairwise comparisons between criteria; and (3) obtain the relative ranking of alternatives in relation to prioritized criteria (Saaty 1988).

#### 2.2.1 Identifying and Organizing Decision Objectives, Criteria, and Alternatives Into the AHP Hierarchy

**FIGURE 2:**
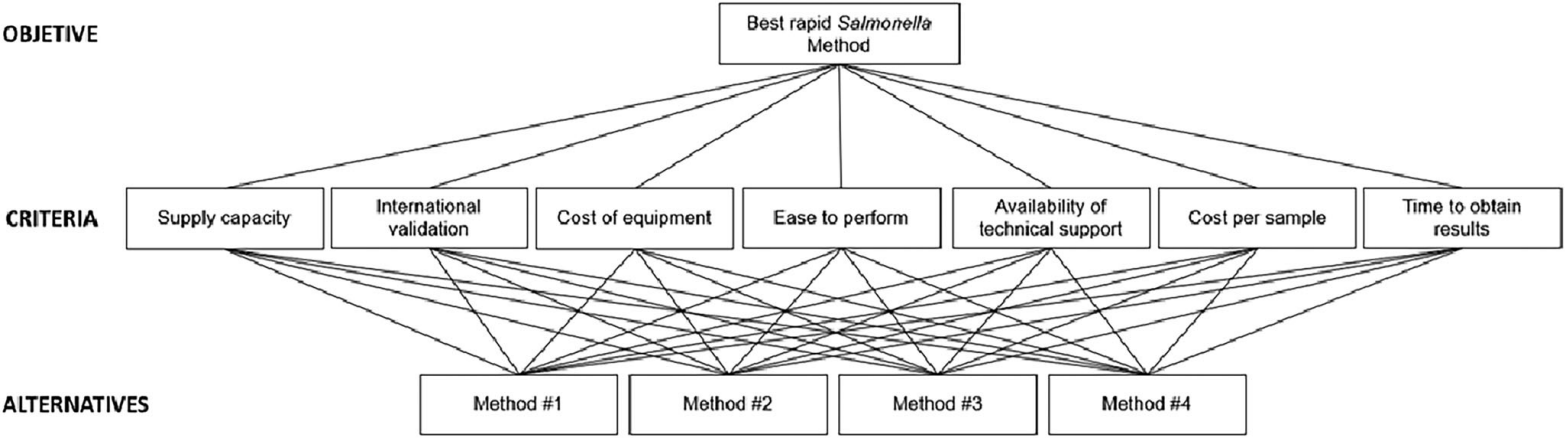
Hierarchical structure of the analytic hierarchy process (AHP) for evaluation of rapid *Salmonella* detection methods.

As a first step, the hierarchical structure of the AHP for the case under study was built (Figure 2) (Saaty 1987, 1990). This demonstrates the objective of selecting the best method for rapid *Salmonella* detection at the top of the hierarchy. At the level below, we list the seven criteria to be evaluated using the pairwise AHP model. At the bottom, we placed the four rapid methods as alternatives that were evaluated in relation to each of the seven criteria.

#### 2.2.2 Pairwise Comparisons of Criteria

As a second step of AHP, a paired comparison of each criterion was performed by the same four experts using the AHPWEB software (2022‐04‐20: Alpha version) (Françozo et al. 2023). In this step, the evaluators analyzed the extent to which criterion A is more important than criterion B, and so on, according to the Fundamental Scale of Saaty (Table 2) (Saaty 1987, 1990, 2013). The questionnaire used for data collection is provided as Supporting Information.

**TABLE 2:** Fundamental scale of Saaty.

Intensity of importance on an absolute scale	Definition	Explanation
1	Equal importance	Two activities contribute equally to the objective
3	Moderate importance of one over another	Experience and judgment slightly favor one activity over another
5	Essential or strong importance	Experience and judgment strongly favor one activity over another
7	Very strong importance	An activity is strongly favored, and its dominance demonstrated in practice
9	Extreme importance	The evidence favoring one activity over another is of the highest possible order of affirmation
2, 4, 6, 8	Intermediate values between the two adjacent judgments	When compromise is needed
Reciprocals of above	If activity *i* has one of the above numbers assigned to it when compared with activity *j*, then *j* has the reciprocal value when compared with *i*	This is logical assumption
Rationals	Ratios arising from the scale	If consistency were to be forced by obtaining numerical values to span the matrix

*Source*: Adapted from Saaty (1990, 2013).

The AHPWEB software (2022‐04‐20: Alpha version) (Françozo et al. 2023) provides a ranking of the criteria priorities considering the individual paired evaluations of each expert, calculated by

$$W_{i}=\left(1/n\right)\times \sum_{j=1}^{n}\left(a_{\mathrm{ij}}/\Sigma a_{\mathrm{ij}}\right)$$

where *W_i_* is the priority (weight) of criterion *i*; *n* is the number of criteria; *a_ij_* is the comparison value of criterion *i* relative to criterion *j*; Σ*a_ij_* is the sum of values in column *j*; Σ is the summation from *j = *1 to *n*.

The aggregation of individual judgments (AIJ) of the criteria were calculated in the software using the geometric mean (Forman and Peniwati 1998), given by

$$J_{g}\left(k,l\right)=\prod_{i=1}^{n}J_{i}{\left(k,l\right)}^{W_{i}}$$

where *J_g_* (*k,l*) is the group judgment of the relative importance of factors *k* and *l*, *J_i_* (*k,l*) is the individual i's judgments of the relative importance of factors *k* and *l*, and *W_i_* is the weight of individual decision‐makers *i*, where the sum of *W_i_* of all decision‐makers is equal to 1.

#### 2.2.3 Ranking of the Evaluated Alternatives

In the third step of AHP, each alternative rapid *Salmonella* detection method was analyzed by absolute measurement, first by the individual judgment of each expert and then by aggregating their judgments.

In the absolute measurement, alternatives were ranked based on how well their characteristics aligned with each prioritized criterion (Saaty 1987, 1990). Each alternative was scored from 0 to 3 for each criterion, in order to reflect the negative or positive impact of attending each criterion (Saaty 1980). These scores were then multiplied by the priority vector of the respective criteria and summed to generate a ratio scale score for the alternatives. Finally, the alternative scores were normalized (Saaty 1987).

The absolute measurement is given by

$$T\left(ai\right)=\sum \left(w_{j}\times s_{ij}\right)$$

where *T*(*ai*) is the total score for alternative *i*; *w_j_* is the weight of criterion *j*; *s_ij_* is the score of alternative *i* with respect to criterion *j*.

To express these totals as final priorities, we normalized the column of totals by dividing each value by the sum of all the values:

$$X_{n}=T\left(ai\right)/\sum T\left(ai\right)$$

where *X_n_* is the normalized value; *T*(*ai*) is the original value (the “total” before normalization); Σ *T*(*ai*) is the sum of all the original values (the sum of all the “totals”).

The AHPWEB software (2022‐04‐20: Alpha version) (Françozo et al. 2023) also provided the aggregation of individual priorities (AIP) using arithmetic means for the alternatives. The AIP with arithmetic means was then validated using geometric means, which showed the same ranking outcome.

The geometric mean approach is preferred because it is recognized to be more consistent (Forman and Peniwati 1998). The geometric mean uses the product operator (∏) to multiply the individual priorities raised by the power of their respective weights, rather than summing the weighted priorities, as in the arithmetic mean formula.

The weighted geometric mean of priorities is given by

$$\begin{array}{c} P_{g}\left(A_{j}\right)=\prod_{i=1}^{n}P_{i}{\left(A_{j}\right)}^{\mathrm{Wi}} \end{array}$$

where *P_g_* (*A_j_*) is the group priority of alternative *j*; *∏* is the product operator from *I = *1 to *n*; *P_i_* (*A_j_*) is the individual i's priority of alternative *j*; *W_i_* is the weight of individual *i*; Σ*w_i_ = *1 (the sum of weights equals 1); *n* is the number of decision‐makers.

The weighted arithmetic mean of priorities is given by

$$\begin{array}{c} P_{g}\left(A_{j}\right)=\sum_{i=1}^{n}W_{i}P_{i}\left(A_{j}\right) \end{array}$$

where *P_g_* (*A_j_*) is the group priority of alternative *j*; Σ is the summation operator from * = *1 to *n*; *P_i_* (*A_j_*) is the individual i's priority of alternative *j*; *W_i_* is the weight of individual *i*; Σ*w_i_ = *1 (the sum of weights equals 1); *n* is the number of decision‐makers.

#### 2.2.4 Evaluation of the Robustness of RBA and AHP Results Through Expanded Expert Panel

The four experts who conducted the RBA initially applied the AHP methodology. To evaluate the robustness of the RBA and AHP results and provide an in‐depth analysis requested by the RBA, a larger expert panel was assembled with 19 professionals, including seven laboratory managers from large‐scale poultry companies, nine experienced lab technicians responsible for rapid method testing and selection in large poultry companies, and three experts from assay manufacturers. These professionals answered a Google Forms questionnaire, ranking their opinions on the importance of each of the seven criteria using a numerical preference scale (Table 3). In this numerical preference scale, each number represents a level of importance: irrelevant (1), moderately important (3), important (5), very important (7), or essential (9). The panelists were unable to select a value among those listed. Additionally, the larger expert panel could mention on the Google form if there were other criteria that they considered important for choosing the best method for the rapid detection of *Salmonella*. The questionnaire used for data collection is provided as Supporting Information.

**TABLE 3:** Numerical preference scale used by 19 experts to express their opinion about the importance of each criterion of the rapid *Salmonella* detection methods.

Numerical preference scale	Definition
1	Irrelevant
3	Moderately important
5	Important
7	Very important
9	Essential

**FIGURE 3:**
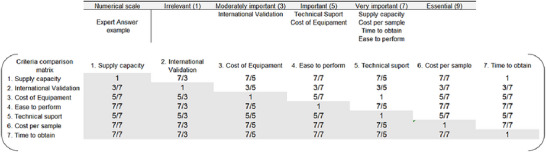
Example of a pairwise comparison matrix completed based on an expert's responses using the numerical preference scale.

As the AHP method requires comparisons between pairs of criteria and the larger panel of experts (*n* = 19) evaluated their criteria using the numerical preference scale (Table 3), the AHP method was adapted by filling in the comparison matrix in Excel with the individual numerical responses. Figure 3 illustrates the pairwise comparison matrix based on the responses of one of the experts using the numerical preference scale presented in Table 3. As an example, if the expert classified supply capacity as very important (7) and international validation as moderately important (3), the pairwise comparison between the row supply capacity versus the column international validation would appear as the fraction “7/3.”

Besides, using Excel, the criteria priorities pointed by the larger panel were identified by AIJ with geometric average, as well as the final alternatives ranking of the larger group decision was shown by AIP with geometric average (Forman and Peniwati 1998). The decision matrices and the AHP calculations are available in the Supporting Information.

In the two expert panels conducted in this study, all decision‐makers were considered to have the same weight of importance in the AHP decision‐making process (Forman and Peniwati 1998).

#### 2.2.5 AHP Sensitivity Analysis

Finally, a sensitivity analysis was performed. In this step, inputs such as criteria weights or performance scores of alternatives are changed to analyze how the results would change and whether the final decision aligns with the overall framework (Dunn 2015; Saaty and Vargas 2012). In the sensitivity test of this study, Method 4 was selected, and its characteristics were adjusted to create different scenarios for reanalysis using the previously ranked criteria.

### 2.3 Statistical Analysis

Statistical analyses were performed using the AHP results. The variables were described by means and standard deviations (SD) or medians and ranges of variation depending on the distribution of the variable. Histograms and the Shapiro–Wilk tests were used to check for normality.

Generalized linear models (GLM) compared criteria and methods, using linear models for variables with a normal distribution and Tweedie with log transformation for those with an asymmetric distribution. To complement this analysis, the Bonferroni test was applied to correct multiple comparisons.

The expert panels were compared using Student's *t*‐test for independent samples (normal distribution) or the Mann–Whitney test (asymmetric distribution).

The significance level was set at 5% (*p* < 0.05). Analyses used SPSS v27.

## 3 Results and Discussion

### 3.1 RBA Results

#### 3.1.1 Risk Profile

Table 4 contains a description of the FSI evaluated using the RBA model, that is, the use of rapid *Salmonella* detection methods in large‐scale poultry industries in Brazil.

**TABLE 4:** Risk profile of the food safety issues (FSI): Use of rapid *Salmonella* detection methods in large‐scale poultry companies in Brazil.

Hazard of concern	*Salmonella* spp.
Description of the food safety issue (FSI)	Microbiological testing of poultry products is necessary to verify the correct implementation of food safety management systems (FSMS), legislation compliance, and the safety of products before trading. In Brazil, *Salmonella* testing is required in poultry farms (Brasil 2016), fresh poultry meat, and poultry products (Brasil 2022). The current technology used in poultry industries does not allow the complete elimination of *Salmonella* in all poultry meat produced worldwide (Hessel et al. 2022). Consequently, *Salmonella* can be found in poultry products, but the prevalence, usually determined in 25 g samples, depends on the appropriate control measures implemented by the FSMS. *Salmonella* control is complex in large poultry producing countries. Brazil is the biggest exporter and the second largest producer of poultry meat in the world. Brazilian industries put a lot of effort into controlling *Salmonella*, because this microorganism is part of the natural poultry microbiota, making it challenging to prevent *Salmonella* in farms and cross‐contamination inside industrial plants. The current microbiological criterion for fresh poultry meat commercialized in Brazil is *n* = 5, *c* = 0 for *Salmonella* Enteritidis and *Salmonella* Typhimurium in 25 g samples (Brasil 2022). The same criteria are required to export poultry products to the European Union (CE 2073/2005) (European Union 2005). Other *Salmonella* spp. criteria can be required by some of the 150 importers of Brazilian poultry meat. Large slaughterhouses are considered those that slaughter 100 thousand to 200 thousand poultry/day, and, according to current Brazilian legislation, these establishments can have up to 12 positive samples for *Salmonella* spp. in a cycle of 51 samples. Positive batches for *Salmonella* Enteritidis and *Salmonella* Typhimurium must be slaughtered separately from the other batches, followed by immediate cleaning and disinfection of the facilities and equipment. These batches also need to be submitted to heat treatment, guaranteeing the elimination of *Salmonella* (Brasil 2016). To attend to these regulations and other microbiological criteria requested by FSMS and importers, it is necessary to carry out several *Salmonella* detection tests, and it is not feasible using traditional methods because they are time‐consuming and can be even more expensive than rapid methods, considering the need of preparing, sterilizing, analyzing samples, and washing materials. Rapid *Salmonella* detection methods have been used by large‐scale companies because they are faster than traditional methods, allowing the analysis of many more samples in less space and by fewer staff. Rapid methods are especially used for the screening of poultry meat samples, allowing the delivery of *Salmonella* negative batches in less time than when analyzed by conventional methods. In Brazil, large companies usually test all batches before they are delivered or shipped. Although a traditional method will need 3–5 days for a *Salmonella* negative result, rapid methods will produce negative results in approximately 20–34 h or less. The time to obtain results is highly dependent on the enrichment step that is required to recover injured cells and increase the number of *Salmonella* to a detectable level. Enrichment steps greater than 8–9 h result in at least 2 working days for *Salmonella* analysis, increasing costs and reducing benefits. Immunoenzymatic and molecular methods seem to be the most frequently used methods in large‐scale poultry industries, requiring approximately 18–24 h of enrichment, and assay runs of 1–3 h. Other methods have shorter enrichment times and are able to detect *Salmonella* in approximately 9 h (Aquino et al. 2021; Nguyen et al. 2020). Independent of the rapid method used, positive results need to be confirmed by culture, biochemical, and serological testing, resulting in approximately one additional week of analysis. There are several available rapid *Salmonella* detection methods, and their characteristics have to be considered before their adoption by laboratories. Some factors to be observed are the accuracy, cost per sample, international validation, the easiness to perform, the necessity and cost of equipment, availability of technical support, supply capacity, the time to obtain results, and the indirect costs in poultry production or distribution due to waiting for the results. Typically, laboratories of large‐scale poultry companies have already implemented one or more rapid methods for *Salmonella* detection. Once implemented, replacing one rapid method with another should be carefully evaluated to ensure that the benefits of the new method outweigh the costs and risks to the company. For example, new methods generally need to be internally validated, employees have to be trained, the method has to be added to the companies’ documents, and internal audits of the new method have to be carried out
Description of the hazard, the severity of clinical manifestations, and its probability to be found in food	Salmonellosis is a serious disease (International Commission on Microbiological Specifications for Foods 2018), causing diarrhea, vomiting, and sometimes fever that usually lasts for 3–5 days. The illness is one of the most important foodborne diseases worldwide. Hospitalization may occur in some cases, but sequelae and deaths are rare. *Salmonella* is also a serious economic problem when found in poultry meat and poultry products because it may cause recalls and the interruption of internal commercialization and exports We considered medium the probability of finding *Salmonella* in Brazilian poultry meat, because the results of the meta‐analysis carried out by Hessel et al. (2022) demonstrated that the prevalence of *Salmonella* in poultry meat was 14.9%, tested in 25 g samples. The *Salmonella* prevalence found in other large‐scale poultry producers was higher, whereas lower prevalences were identified in smaller producers
Economic impact of the use of rapid methods	In this study, we assumed that one large‐scale poultry company slaughters from 100 thousand to 200 thousand poultry/day and tests from 15 to 50 samples/day for *Salmonella*, i.e. 450–1500 samples/month. The assumed costs of rapid detection methods usually found in laboratories of Brazilian poultry companies vary from $4 to $5. The cost per sample is an estimate carried out by the authors of this study and may not reflect the reality found in the market. These costs can vary according to the number of tests done by companies and dollar variation in Brazil. However, the proportion among the prices was assumed to be correct. On the basis of that, it is estimated that a large poultry company spends an average of $2025–$6750 per month on testing costs. We assumed that the salary of one Brazilian laboratory employee is $900–$1100 The economic impact of using rapid *Salmonella* detection methods also was measured considering the costs to keep refrigerated or frozen poultry meat inside loaded trucks waiting for *Salmonella* analysis results. In Brazil, poultry products are processed and then stored in warehouses before being shipped out. Due to the volume of production, there are also distribution centers along the supply chain. However, along the logistics chain, chilled or frozen poultry meat can also be stored temporarily in trucks, while awaiting authorization for shipment within Brazil or export. We use this truck storage scenario as it is the last stage before the products reach the market and is often when *Salmonella* detection tests are being finalized. The cost to store one loaded truck containing 24 t of poultry meat or poultry products in Brazil for the first 24 h was estimated in $3.67 per hour. If the food stays more than 24 h, the cost per hour was estimated to increase to $7.33. Bigger trucks containing 30 t of poultry meat would cost $4.78 per hour, in the first 24 h and $9.57 per hour, after 24 h of storage. As an example, the waiting time for a *Salmonella*‐negative result using traditional methods would take from $439.92 to $791.76 per 24 t of poultry, while the time for a rapid test would cost much less, from $33.03 to $161.38 for the same particular truck. These costs can be very high considering the annual poultry meat production of 14.5 million tons in Brazil, with more than 5 million tons exported in 2023 (Associação Brasileira de Proteína Animal 2024). This amount of exportation generated almost 10 billion dollars in revenues in 2023 (CEPEA/ESALQ 2024). Thus, reliable rapid tests have a critical role in this market, as a false‐positive S*almonella* result could have strong impacts on costs related to blocking exports or increasing storage time until the biochemical and serological confirmation testing to release the cargo to the market
Control measure to the FSI	Choose the best rapid *Salmonella* detection assay according to its characteristics

*Source*: Adapted from Tondo and Gonçalves (2021).

#### 3.1.2 RBA Risk Ranking

Risk ranking was used to determine the urgency of actions to control the hazard, assuming that higher risks should be controlled faster (Tondo and Gonçalves 2021). On the basis of the FSI description (Table 4), *Salmonella* spp. were identified as a hazard of concern. The risk associated with *Salmonella* was ranked according to the severity and likelihood categories of the risk‐ranking matrix used in the RBA model (Tondo and Gonçalves 2021) (Table 5). On the basis of this, we ranked the *Salmonella* risk in poultry meat as medium, considering that salmonellosis is a serious disease and the likelihood of finding *Salmonella* in fresh poultry meat in Brazil is medium (14.9%) (Hessel et al. 2022). According to the RBA, medium‐risk demand control measures are adopted in the short‐ to medium‐term.

**TABLE 5:** Risk ranking matrix according to severity and likelihood of the hazard involved in food safety issues (FSI) as analyzed by risk–benefit analysis.

Severity	Likelihood				
	Very low (1)	Low (2)	Medium (3)	High (4)	Very high (5)
Low (1)	1	2	3	4	5
Moderate (2)	2	4	6	8	10
Serious (3)	3	6	9	12	15
Severe (4)	4	8	12	16	20
Very severe (5)	5	10	15	20	25

*Note*: The greater the risk identified, the greater the urgency of control. White squares = low risk: Control measures can be adopted in the long term; Gray squares = medium risk: Control measures could be adopted in the mid‐term; Dark gray squares = high risk: Control measures should be adopted in the short term or immediately.

*Source*: Adapted from Tondo and Gonçalves (2021).

#### 3.1.3 RBA Benefits and Costs

On the basis of the risk profile and risk ranking, four food safety experts analyzed the benefits and costs (Table 1) of adopting the most adequate rapid *Salmonella* detection method in poultry companies, which was identified as the control measure of the FSI. The analyses considered the seven criteria listed by the four experts as the most relevant for evaluating rapid *Salmonella* detection tests.

According to the risk profile information (Table 4), although rapid methods can generate negative results for *Salmonella* in 9–34 h, traditional methods require at least 3 days to generate similar results, that is, at least 2 days more. In addition, *Salmonella*‐positive results must be confirmed by culture, biochemical, and serological testing, which require approximately one additional week. On the basis of these results, the food safety experts considered that the use of rapid *Salmonella* detection methods brings high to very high benefits to large poultry companies, as the detection can be performed in less time, with a higher number of samples and less labor force, considering the same laboratory space used for traditional methods. The costs of rapid assays were considered low to high for large poultry companies, depending on whether the equipment used to run rapid methods was on loan. Therefore, the RBA recommended the use of rapid methods because the benefits are high to very high and costs are low to high for large poultry companies (Table 1).

According to the food safety experts, supply capacity was classified as an essential characteristic of rapid detection methods; otherwise, companies would not have the necessary assays to carry out analyses or the confidence to implement a method if the required supplies are lacking during its execution. This need has been highlighted in the COVID‐19 pandemic, which has raised awareness of the need for reliable suppliers to ensure the consistent availability of essential test materials. It has become clear that the ability to supply laboratory supplies is essential, as it directly affects the ability to carry out tests effectively. Disruptions in the supply chain can delay diagnostic tests and their results compromising food safety programs and substantially increasing costs. Thus, assays with sufficient supply capacity throughout the year, especially with rapid deliveries, bring very high benefits to poultry companies while keeping costs under control.

International validation of rapid detection assays was considered a very important criterion because laboratories must use methods that meet the proposed purposes, ensuring valid results that contribute to controlling the risk of salmonellosis. To accomplish this purpose, the Brazilian Ministry of Agriculture and Livestock (MAPA) published in 2023 that the following elements are essential to ensure the quality of issued results: verification of method performance, calibration of critical equipment, use of reference materials, proficiency studies, training of laboratory personnel, internal and external audits, and method validations are essential to ensure the quality of the results issued (Brasil 2023). On the basis of this document, the detection methods used in laboratories of exporter poultry companies in Brazil should be validated and recognized internationally by the AOAC, AFNOR, ISO Methods, MicroVal, NordVal, and among others (Brasil 2023). International validation was not considered an essential criterion for rapid detection assays because Brazilian poultry companies could use alternative methods (Brasil 2023). However, the adoption of such methods may result in increased testing costs and time, thus reducing benefits. Moreover, importers of Brazilian poultry meat may not accept the use of alternative methods, causing companies to prefer and adopt internationally validated methods. Hence, assays with international validations bring high benefits to poultry companies, facilitating the recognition of the method in internal and external markets.

The availability of technical support was considered a very important criterion by experts, even more important than ease to perform, especially in the implementation phase of a new methodology in the company's laboratory, when technical support brings very high benefits and avoids error costs. However, the experts also rated its importance as very relevant for the methods already in use, because critical or urgent situations related to the assays require rapid and accurate technical support from suppliers. Considering these, Method 2 may not be prioritized as a choice when compared with Methods 1 and 3 because it shows lower technical support than the other methods.

The criterion “ease to perform” was classified as important by the experts because it brings high benefits, such as less training time for the laboratorians, higher productivity, and lower risk of human error when carrying out the tests. Therefore, in general, the total cost will decrease because of the lower reagent consumption, fewer working hours, and shorter processing times. However, it was considered less important than the criteria discussed above.

The cost per sample was considered a moderately important criterion for rapid *Salmonella* detection methods, although lower prices bring higher benefits to poultry companies. The assay costs depend on the number of samples analyzed in the laboratories. Assuming that large poultry companies analyze 450–1500 *Salmonella* samples per month, the costs can range from $2025 to $6750 per month, as discussed in the risk profile (Table 4). Laboratory managers indicated that the costs were reasonable (low to medium) and that price differences among assays were not the most important factor in selecting a rapid method. Given this information, the cost per sample was classified as a moderately important criterion. Beyond that, the difference in prices can be more relevant depending on the number of tests purchased yearly and the contracts done with the assay's manufacturing companies; thus, commercial proposals are important to reduce costs.

The low equipment cost of the new rapid methods is another important feature desired by the consulted experts. For Methods 1–3, the equipment costs were considered low (Table 6) because assay manufacturers lend equipment to the poultry laboratories that purchase their assays. This loan procedure brings high benefits to companies and, based on that, the RBA recommends that it be required. However, because assay manufacturers usually lend equipment, this criterion was considered irrelevant for poultry companies when choosing rapid methods. Materials, such as plastic bags, pipettes, and disposable materials, are not lent, but their costs were considered low, and laboratories generally already have these materials. Depending on the method, laboratories need to buy incubators to grow microorganisms at specific temperatures, and their costs were also considered low because, if needed, the equipment will be bought and will last for a long time. Therefore, even with additional costs, the costs would be low, and the benefits would be at least high, resulting in the RBA model recommending the adoption of rapid methods that require these laboratory materials. Even assuming that Method 4 would require higher equipment costs (this was the worst scenario assumed by the authors because this method is not yet available in the market), the benefits would be at least high, and the RBA would recommend the adoption of all rapid methods, including Method 4.

**TABLE 6:** Prioritization of the seven main criteria evaluated by the risk–benefit analysis (RBA) and the characteristics of four rapid *Salmonella* detection methods.

	Criteria							
		Supply capacity	International validation	Cost of equipment	Ease to perform	Availability of technical support	Cost per sample^a^	Time to obtain *Salmonella* results
	Prioritization^b^	Essential	Very important	Irrelevant	Important	Very important	Moderately important	Very important
Assay characteristics	Method 1	+++	AOAC, AFNOR	Low	+++	+++	5	20–26 h per 96 samples
	Method 2	+++	AOAC, AFNOR	Low	++	++	5	30–34 h per 96 samples
	Method 3	+++	AOAC, AFNOR	Low	++	+++	4	20–26 h per 30 samples
	Method 4	NI	AOAC	High^c^	+++	NI	6^d^	9–10 h per 96 samples

Abbreviation: NI, not identified.

^a^The cost per sample in American dollars ($) is an estimation carried out by the authors of this study and may not reflect the reality found in the market.

^b^Prioritization of seven criteria evaluated by the four food safety experts.

^c^These costs were assumed, considering that the equipment for a faster method would not be delivered on loan.

^d^This cost was created, considering that a faster method would cost 20% more than other methods.

+, ++, +++: Qualitative scale indicating the degree to which the method meets the criterion; (+) low; (++) moderate; (+++) high.

The time taken to obtain results was considered a very important factor in rapid detection assays and was identified as even more important than the cost per sample. According to food safety experts, this characteristic could justify the substitution of a rapid method with a faster method because of the economic impacts on production and distribution costs. For example, fresh poultry meat and poultry products must be stored in cold chambers or inside refrigerated trucks while samples are being tested. In Brazil, poultry products of large‐scale companies are generally not released to the market until *Salmonella* results are negative or in compliance with the performance standards established by the government. *Salmonella*‐positive results must be confirmed using traditional methods, increasing the time to obtain final results and, consequently, the storage time and costs before product release (detailed in FSI, Table 4). The information in Table 6 demonstrates that Methods 1–3 can generate results within approximately 24 h. Assuming this period as the time that products are stored while they are waiting for release to the market, one loaded truck containing 24 t of poultry product would cost $88.08, while the next 24 h would cost $175.92. Progressive pricing encourages faster use because the space is designed for rapid turnover, not long‐term storage. The longer a truck stays at a dock, the more it can affect the scheduling of other trucks, creating bottlenecks in the supply chain. Therefore, if this refrigerated truck has to wait for the confirmation of *Salmonella* results by traditional methods (at least three additional days for testing) after a rapid test‐positive result, the total storage time would cost $615.84 per 24 t‐truck. Bigger trucks containing 30 t of poultry meat would cost $114.72 (first day) plus $229.68 per additional day, resulting in $803.76, for four storage days. Thus, the time to obtain results is a very important and strategic criterion when choosing a rapid *Salmonella* test and should be carefully considered by poultry companies. On the basis of this information, the faster the method, the greater the benefits it provides, and the lower the costs. Therefore, RBA recommends the adoption of faster methods.

In this sense, assay Method 4 would bring very high benefits to the companies because this method can identify *Salmonella* in 9 h. According to the experts, the adoption of this method could be advantageous even if the cost per sample was higher than the other rapid methods and the cost of equipment being high, justifying its implementation as long as the supplier has adequate supply capacity, international validation, and availability of technical support.

Therefore, in response to the first question addressed by this study, which is, What are the benefits and risks of using rapid *Salmonella* detection assays in large‐scale poultry industries? The RBA considered that the adoption of these rapid methods is clearly advantageous, bringing high to very high benefits to companies with low to high costs, and recommending their implementation (Table 1).

However, concerning the second question of the study, what are the benefits and risks of changing one rapid method implemented by another? RBA was unable to identify the benefits of changing one well‐known method to another because their characteristics were quite similar, as described in Table 6. This change can result in low benefits or even be disadvantageous (no benefit), as the adoption of a new rapid method by a poultry company requires validation of the new method for each food matrix analyzed in the laboratory, description of the new method to include it in the certification scopes, new calibrations, training people in all laboratories of the food company, and carrying out internal and external audits, which could result in additional costs. Moreover, the intangible costs of internal change, such as employee learning time, resistance to change, errors in carrying out the new test, and their consequences, resulted in low benefits. Low benefits with low, medium, high, or very high costs are identified in the RBA as an uncertainty zone, meaning that more information is required to decide whether to take action (Table 1). On the basis of this, the AHP methodology was applied with the same four food safety experts (expert panel 1) and later with a group of 19 experts (expert panel 2), composed of laboratory managers and assay experts from assay manufacturing companies. The results of the RBA and AHP were then compared.

### 3.2 AHP Results

#### 3.2.1 AHP Criteria Ranking

Using the AHP method, the seven criteria listed for choosing the rapid methods were ranked in terms of importance by pairwise comparison for each expert panel. The judgment of the pairwise comparisons was considered consistent, as the consistency ratio (CR) resulting from each of the expert's analysis was <0.006.

**FIGURE 4:**
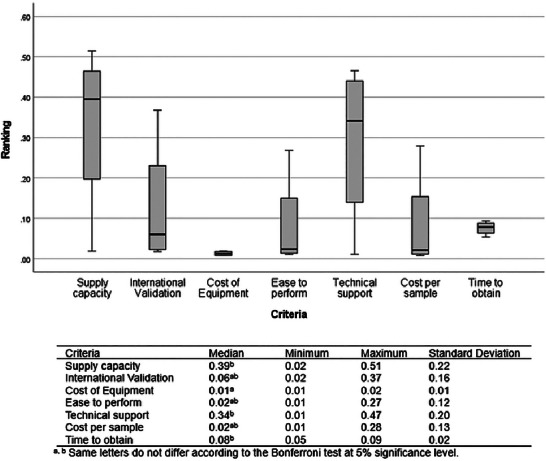
Criteria ranking based on aggregated individual judgments (AIJ) of expert panel 1 (*n* = 4).

The AIJ of expert panel 1 (*n* = 4) showed the following priority ranking of medians: supply capacity (0.39), technical support (0.34), time to obtain results (0.08), international validation (0.06), ease to perform and cost per sample (0.02), and cost of equipment (0.01) (Figure 4). Supply capacity, technical support, and time to obtain results were ranked significantly more important than the cost of equipment (Bonferroni test, *p* < 0.001). The other criteria did not differ significantly (Bonferroni test, *p *> 0.05). The absence of a significant difference between the high median values for supply capacity (0.39) and technical support (0.34) and the lower median values for international validation (0.06), ease to perform, and cost per sample (0.02) may be explained by the high standard deviation of the small sample size of expert panel 1 (*n* = 4). The preference for different criteria varied among the experts, resulting in a high standard deviation. Only the criteria cost of equipment (min: 0.01; max: 0.02) and time to obtain results (min: 0.05; max: 0.09) showed low variability among the individual expert judgments (Figure 4). The difference in the prioritization of each expert is based on their individual experience and area of activity in the rapid test analysis chain. The expert panel 1 (*n* = 4) was composed of a laboratory manager from a large poultry company, an experienced technical laboratory manager who provides services to the poultry industry, an experienced professor of food microbiology, and an experienced microbiologist responsible for testing and analyzing rapid *Salmonella* detection methods for research purposes. It is important to note that all seven criteria were initially listed in the study by the expert panel 1 as the most important criteria for evaluating the characteristics of rapid tests. Therefore, the analysis of the level of importance between them can be challenging, with the preference between one and another varying slightly according to individual experience and area of activity. The pairwise comparison of the criteria in AHP makes it possible to quantify the greater or lesser importance of one criterion in relation to another for each evaluator. The positive aspect of the AHP method is that the choice of the best alternative will be the one that meets the group's aggregated priorities, bringing objectivity and neutrality to the group's decision‐making.

**FIGURE 5:**
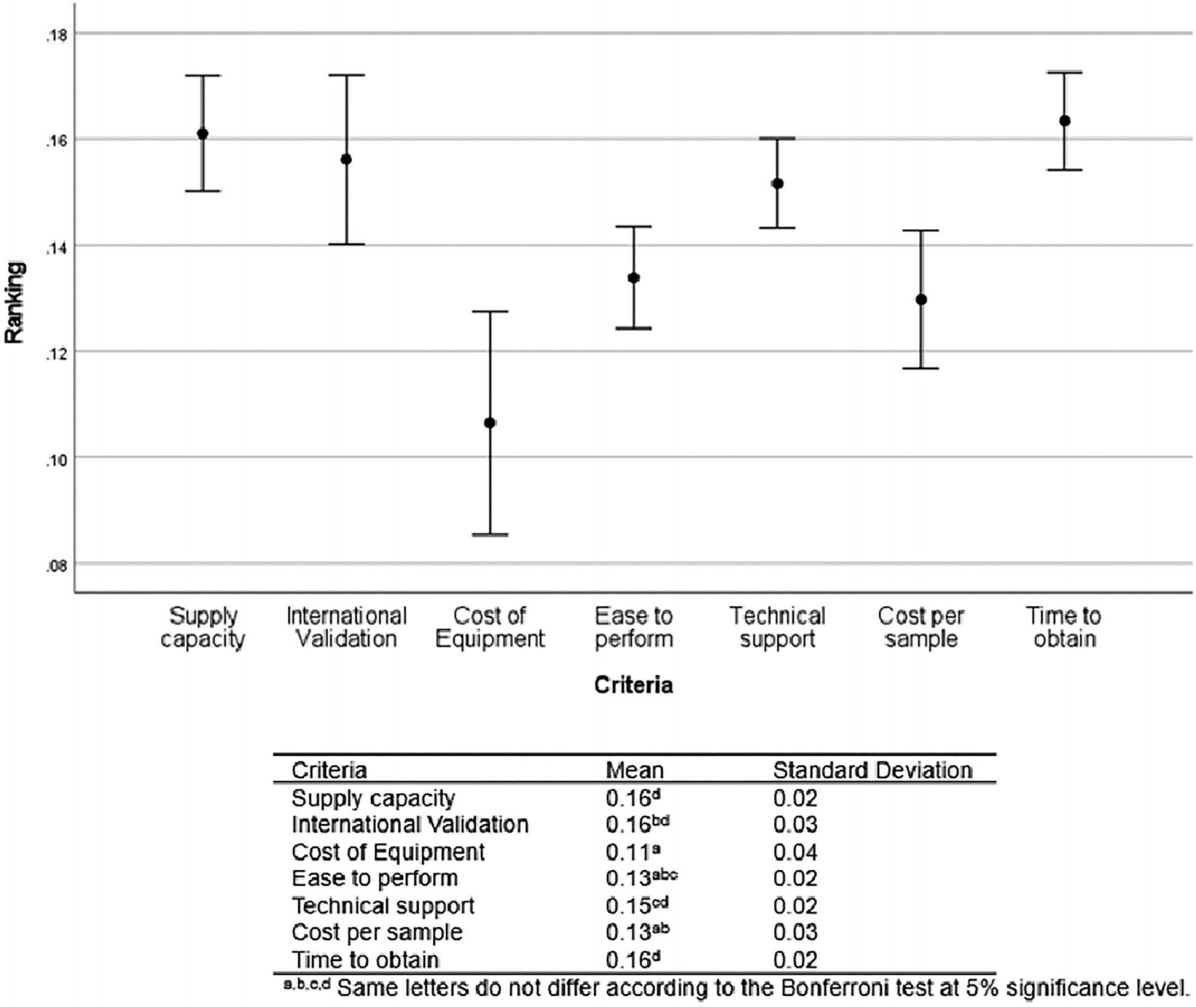
Criteria ranking based on aggregated individual judgments (AIJ) of expert panel 2 (*n* = 19).

The aggregation of the individual judgments of expert panel 2 (*n* = 19) showed the following priority ranking of the means: supply capacity, international validation, time to obtain results (0.16), technical support (0.15), ease to perform and cost per sample (0.13), and cost of equipment (0.11) (Figure 5). Supply capacity, international validation, time to obtain results, and technical support were ranked as significantly more important criteria than the cost of equipment (Bonferroni test, *p* < 0.001) and did not differ significantly from each other (Bonferroni test, *p *> 0.05). The ease to perform and cost per sample did not differ significantly from the cost of equipment or international validation (Bonferroni test, *p *> 0.05) but was ranked significantly less important than supply capacity and time to obtain results (Bonferroni test, *p* < 0.001). Technical support was ranked significantly more important than cost per sample (Bonferroni test, *p* < 0.001) but did not differ significantly from ease to perform (Bonferroni test, *p *> 0.05). The expert panel 2 (*n* = 19) showed a low standard deviation (Figure 5). This may be attributed to the adaptation of the AHP method when collecting responses from the 19 experts using a numerical preference scale (Table 3). More than 65% of these experts classified four of the seven criteria as essential, namely, supply capacity, international validation, time to obtain results, and technical support. These were the four best‐rated criteria that did not differ significantly from one another (Bonferroni test, *p *> 0.05).

**FIGURE 6:**
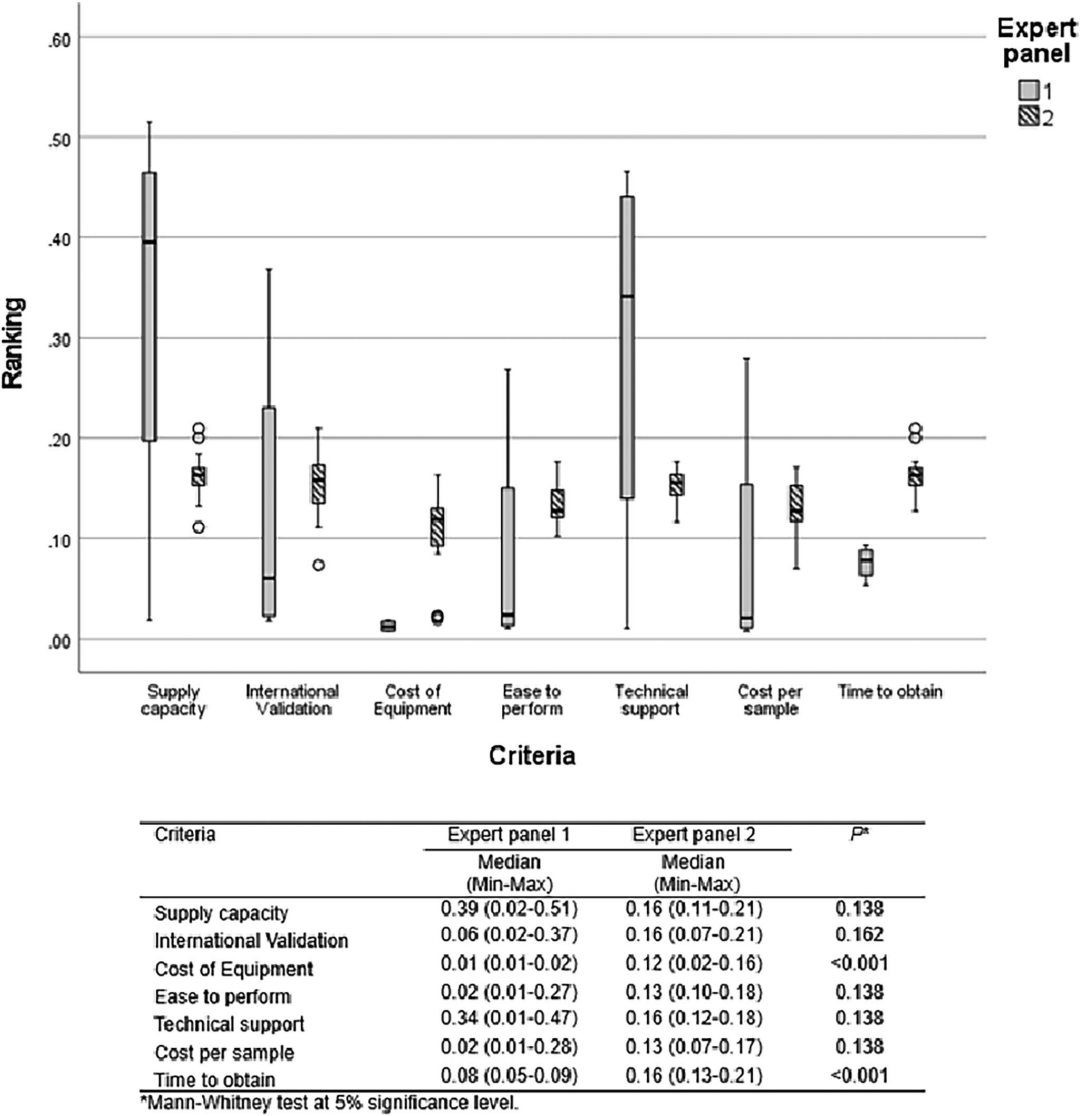
Comparison of criteria ranking between expert panels 1 (*n* = 4) and 2 (*n* = 19).

Figure 6 shows the judgments of both expert panels. There was a significant difference between the medians of the expert panels’ judgments only for the cost of equipment and time to obtain the results (Mann–Whitney, *p* < 0.001). However, for both expert panels, the cost of the equipment criterion was ranked as less relevant, and the time to obtain the results was among the main criteria in the choice of a rapid method.

#### 3.2.2 Comparison of RBA and AHP Results in Prioritizing Criteria

Table 7 shows that in both expert panels, the first four main criteria ranked by AHP when choosing a rapid method for *Salmonella* detection were supply capacity, technical support, time to obtain results, and international validation. Both panels had the same ranking priorities from the fifth to the seventh criterion analyzed: ease to perform, cost per sample, and cost of equipment.

**TABLE 7:** Comparison of the AHP (analytical hierarchy process) and the RBA (risk–benefit analysis) criteria prioritization between expert panels 1 (*n* = 4) and 2 (*n* = 19) for rapid *Salmonella* detection methods.

Criteria ranking	RBA expert panel 1 (prioritization)	AHP expert panel 1 (median)	AHP expert panel 2 (median)
1	Supply capacity (essential)	Supply capacity^b^ (0.39)	Supply capacity^D^ (0.16)
2	Technical support (very important)	Technical support^b^ (0.34)	Time to obtain results^D^ (0.16)
3	Time to obtain results (very important)	Time to obtain results^b^ (0.08)	Intern. validation^BD^ (0.16)
4	Intern. validation (very important)	Intern. validation^ab^ (0.06)	Technical support^CD^ (0.16)
5	Ease to perform (important)	Ease to perform^ab^ (0.02)	Ease to perform^ABC^ (0.13)
6	Cost per sample (moderately important)	Cost per sample^ab^ (0.02)	Cost per sample^AB^ (0.13)
7	Cost of equipment (irrelevant)	Cost of equipment^a^ (0.01)	Cost of equipment^A^ (0.12)

*Note*: Same letters (a, b, A, B, C, D) do not differ according to the Bonferroni test at 5% significance level.

Table 7 also shows the RBA criteria ranking performed by expert panel 1, allowing for a comparison between the AHP and RBA criteria prioritizations. The results of AHP and RBA showed similar rankings for the criteria. The first four criteria prioritized qualitatively by expert panel 1 using RBA as essential (supply capacity) or very important (technical support, time to obtain results, and international validation) were the same as those quantified by AHP for both expert panels. The RBA also classified the cost of equipment as irrelevant, just as the AHP prioritized it last, considering that it is usually given on a loan.

However, in RBA analyses, the ease of performing a rapid test was evaluated as important, and the cost per sample was moderately important. Nevertheless, the AHP revealed no significant differences between these two criteria in the preferences of both expert panels. In the qualitative cost–benefit analyses of the RBA, the expert panel 1 considered that the ease of performing an assay has a direct correlation with the time required to perform the test. A simpler test methodology usually involves fewer preparation steps, fewer manual interventions, less complex sample processing, shorter incubation or reaction times, and a reduced risk of human error. This would result in an overall cost reduction through productivity gains and waste reduction. Overall, the total cost per sample would decrease because of the lower reagent consumption, fewer working hours, and shorter processing times. However, AHP evaluation did not identify that this gain would be significant, maintaining the same degree of priority among the criteria for choosing rapid *Salmonella* detection methods.

#### 3.2.3 AHP Alternative Ranking

Once the priorities of the seven criteria are set in the AHP, the priority of *Salmonella* rapid method alternatives can be determined by aggregating the individual priorities (AIP) for both expert panels.

**FIGURE 7:**
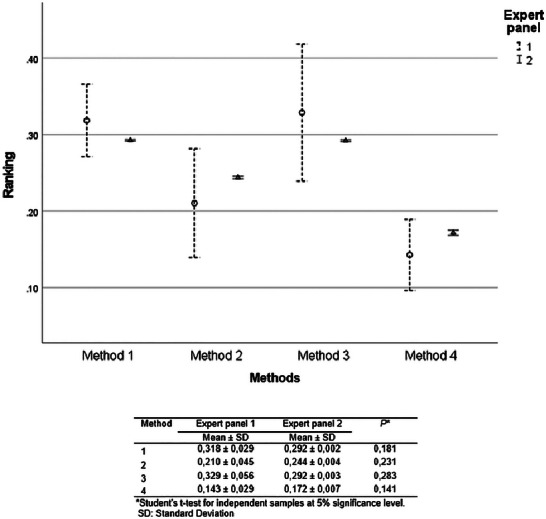
Comparison of *Salmonella* rapid method alternatives ranking between expert panels 1 (*n* = 4) and 2 (*n* = 19) based on aggregated individual priorities (AIP). SD, standard deviations.

There was no statistical difference in the prioritization of the choice of rapid methods between the expert panels using AHP (Student's *t*‐test, *p *> 0.05) (Figure 7).

**FIGURE 8:**
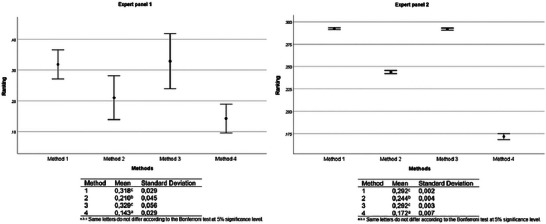
*Salmonella* rapid method alternatives ranking based on aggregated individual priorities (AIP) of expert panels 1 (*n* = 4) and 2 (*n* = 19).

For both expert panels, Methods 1 and 3 were evaluated as equivalent (Bonferroni test, *p *> 0.05), being the preferred choice compared to Methods 2 and 4 (Bonferroni test, *p* < 0.001). However, Method 2 ranked significantly better than Method 4 (Bonferroni test, *p* < 0.001) (Figure 8).

As can be seen in Table 6, Methods 1 and 3 equally met the most important characteristic with a small difference only regarding the easiness to perform, where Method 1 presented a better performance than Method 3. In contrast, Method 3 presented a lower cost per sample than Method 1. These results indicate that the choice of one of the two tests will depend on the commercial proposals made by the assay manufacturers, without prejudice to the results and performance. Method 2 is the next choice because it has a lower availability of technical support (Table 6), which is one of the main criterion pointed out, as shown in Table 7. Moreover, Method 2 takes longer to produce results than Methods 1 and 3. Nonetheless, other criteria may be considered advantageous, in addition to the business proposals, as highlighted by some of the 19 experts consulted. These include specificity, test reliability, an analytical protocol aligned with standard methods such as ISO, the ability to be applied to various food matrices, the capacity to identify different *Salmonella* serotypes, validation for multiple food matrices, the possibility of testing environmental samples, and lower rates of false positives and false negatives.

New Method 4, which presented the best performance in terms of the time required to obtain results (Table 6), was the fourth choice among the evaluated tests (Bonferroni test, *p* < 0.001), as it still did not meet the main criteria listed, such as supply capacity and technical support, because the method is not yet available in the market.

#### 3.2.4 AHP Sensitivity Analysis

**FIGURE 9:**
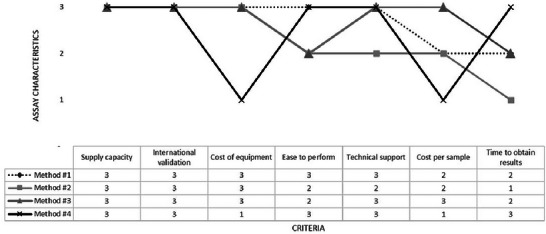
Method 4 meets supply capacity and technical support criteria (Scenario 1) for the analytical hierarchy process (AHP) sensitivity analysis. The *y*‐axis represents the 0–3 level of fulfillment of the method characteristics with respect to the selection criteria.

As a final step of AHP, we proceed with sensitivity analyses. Method 4 was selected to assess two different scenarios. In Scenario 1, we considered that this new method would meet the two criteria of supply capacity and technical support, keeping the estimated cost per sample higher than the others and without offering the equipment on loan (Figure 9).

**FIGURE 10:**
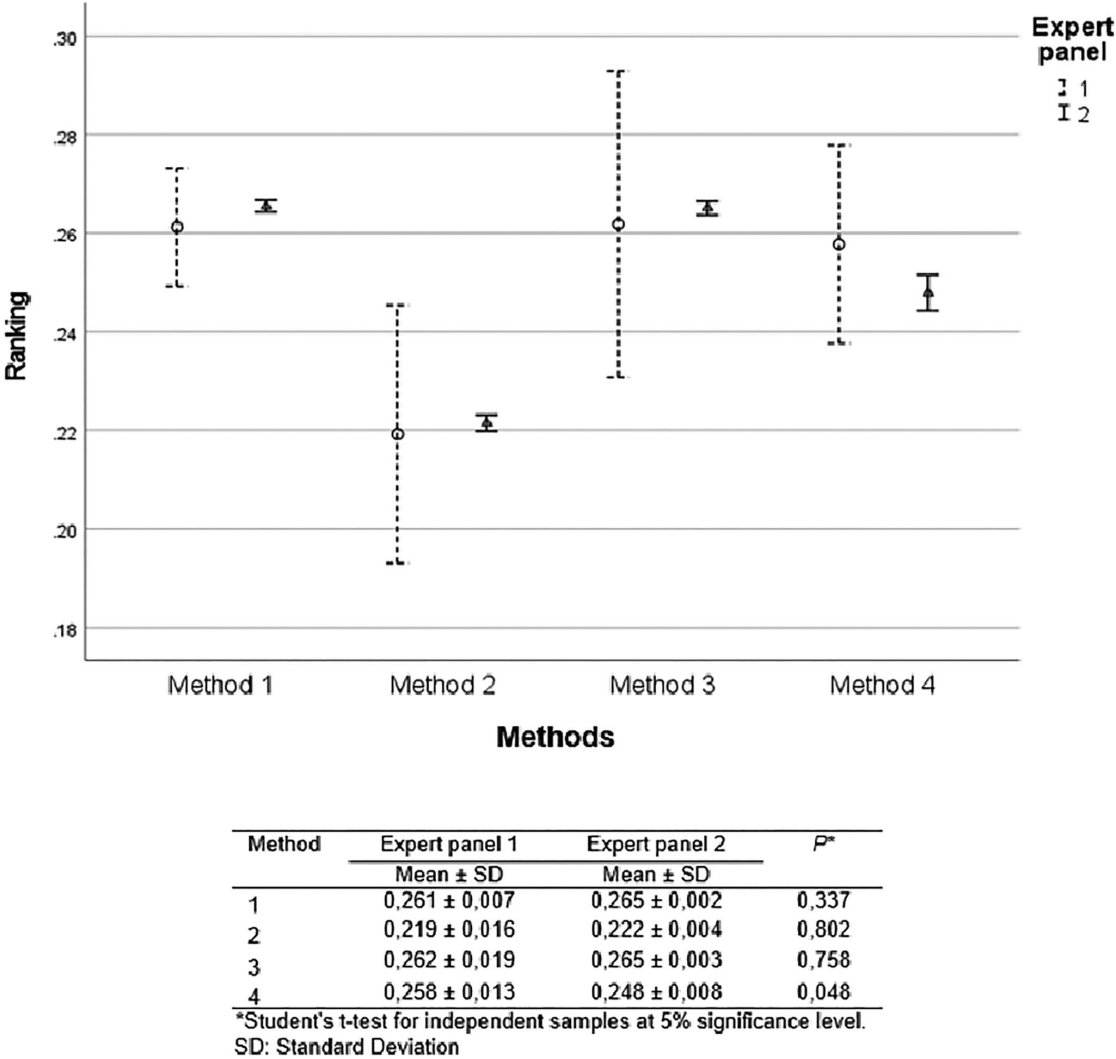
Comparison of *Salmonella* rapid method alternatives ranking between expert panels 1 (*n* = 4) and 2 (*n* = 19) of the sensitivity analysis (Scenario 1) based on aggregated individual priorities (AIP). Scenario 1: Method 4 meets supply capacity and technical support criteria.

In Scenario 1, there was a statistical difference between the expert panels in the classification of Method 4 using AHP (Student's *t*‐test, *p* = 0.048) (Figure 10).

**FIGURE 11:**
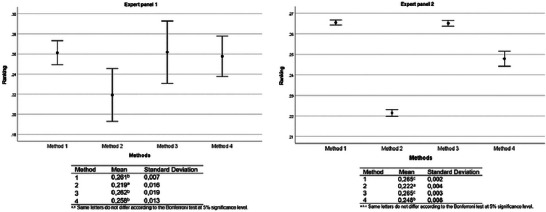
*Salmonella* rapid method alternatives ranking based on aggregated individual priorities (AIP) of expert panels 1 (*n* = 4) and 2 (*n* = 19) for sensitivity analysis (Scenario 1). Scenario 1: Method 4 meets supply capacity and technical support criteria.

For both expert panels in Scenario 1, Method 4 increased in priority ranking by meeting the supply capacity and technical support criteria. For expert panel 1, Method 4 was ranked first, along with Methods 1 and 3, with no statistically significant differences between them (Bonferroni test, *p *> 0.05). However, for expert panel 2, Methods 1 and 3 retained their priority status over Methods 2 and 4 (Bonferroni test, *p* < 0.001). But Method 4 was preferred over Method 2 (Bonferroni test, *p* < 0.001), which would become the last choice, differing significantly from the others (Bonferroni test, *p* < 0.001) for both expert panels (Figure 11). This result confirms the priority of supply capacity, technical support, and time to obtain result criteria in the choice of rapid methods.

**FIGURE 12:**
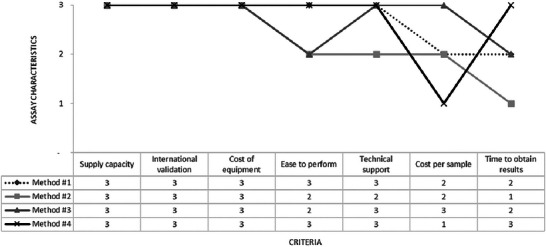
Method 4 meets the supply capacity, technical support, and cost of equipment criteria (Scenario 2) for the analytical hierarchy process (AHP) sensitivity analysis. The *y*‐axis represents the 0–3 level of fulfillment of the method characteristics with respect to the selection criteria.

In Scenario 2 of the sensitivity analysis, it was also considered that the supplier of new Method 4 would provide the equipment on loan, as would other assay manufacturers (Figure 12).

**FIGURE 13:**
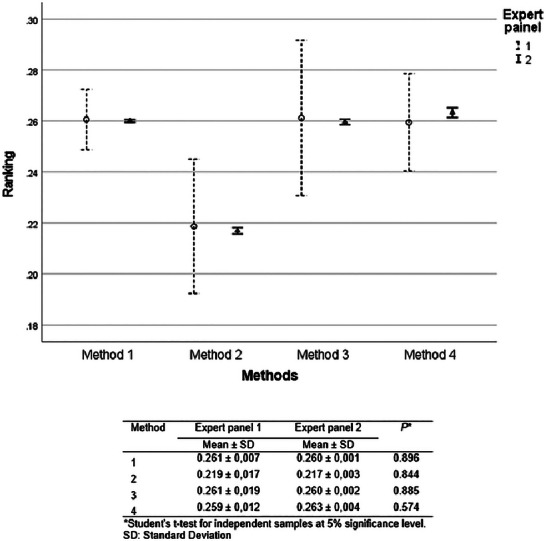
Comparison of *Salmonella* rapid method alternatives ranking between expert panels 1 (*n* = 4) and 2 (*n* = 19) for sensitivity analysis (Scenario 2) based on aggregated individual priorities (AIP). Scenario 2: Method 4 meets the supply capacity, technical support, and cost of equipment criteria.

In Scenario 2, there were no statistical differences between the expert panel choices (Student's *t*‐test, *p *> 0.05), and Method 4 was the first choice for both expert panels (Figure 13).

**FIGURE 14:**
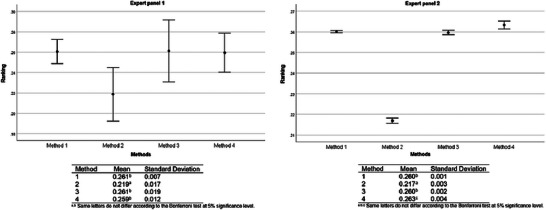
*Salmonella* rapid method alternatives ranking based on aggregated individual priorities (AIP) of expert panels 1 (*n* = 4) and 2 (*n* = 19) for sensitivity analysis (Scenario 2). Scenario 2: Method 4 meets the supply capacity, technical support, and cost of equipment criteria.

For expert panel 1 (*n* = 4) in Scenario 2, Method 4 was ranked equally as Methods 1 and 3 as one of the top alternatives (Bonferroni test, *p *> 0.05). This result is similar to that of the previous sensitivity analysis scenario. In contrast, for expert panel 2 (*n* = 19), Method 4 emerged as the primary alternative of choice among the methods (Bonferroni test, *p* < 0.001). Methods 1 and 3 remained statistically tied in second place (Bonferroni test, *p *> 0.05), whereas Method 2 was ranked last (Bonferroni test, *p* < 0.001) (Figure 14). These results suggest that when rapid methods exhibit similar characteristics in terms of supply capacity, international validation, and equipment loan availability, as well as provide good technical support, a method that can deliver results most quickly may be the preferred choice.

#### 3.2.5 Comparative Analysis of RBA and AHP for Rapid *Salmonella* Detection Method

The AHP results confirmed the robustness of the RBA findings. Both the AHP and RBA analyses indicated that Methods 1 and 3 would be preferred over Method 2 because of their higher technical support and faster time to deliver results.

RBA and AHP also identified that Method 4 could be the best choice for delivering faster results, despite the higher cost per sample and equipment requirements. However, for Method 4 to be selected, it would need to have an adequate supply capacity, international validation, and availability of technical support. Likewise, the AHP sensitivity analysis suggests that when rapid methods exhibit similar characteristics in terms of supply capacity, international validation, and equipment loan availability as well as provide good technical support, a method that can deliver results most quickly may be the preferred choice.

Returning to the RBA analysis with more information provided by the AHP results, it was possible to assess the benefits and risks of changing one implemented rapid method to another (Table 8). The findings indicated that the benefits of changing from a well‐known method to a new faster method outweigh the risks, as long as the faster assay also offers efficient supply capacity, availability of technical support, equipment on loan, and international validations.

**TABLE 8:** Analysis of the benefits and costs of using rapid *Salmonella* detection methods in large‐scale poultry companies in Brazil using the risk benefit analysis (RBA).

Hazard of concern	Risk^a^	Urgency in adopting control measures based on risk^b^	Control measure (CM)	Benefit/cost	Recommendation	Benefit and cost after additional information	Final recommendation
***Salmonella* spp**.	Medium	Short to medium terms	Choose the best rapid *Salmonella* detection assay according to its characteristics	Benefit: high to very high Cost: low to high	Adopt CM	—	—
			Changing one implemented rapid method by another	Benefit: none to low Cost: high to very high	Uncertainty zone^c^. More information is required Apply AHP	Benefit: high to very high Cost: medium to high	Changing the method is beneficial when it delivers faster time to obtain *Salmonella* results, equipment is on loan, international validations and high performance in supply capacity and technical support

Abbreviation: AHP, analytic hierarchy process.

^a^ Low, Medium, or High.

^b^ The greater the risk identified, the greater the urgency of control. Low risk: Control measures can be adopted in the long term. Medium risk: Control measures could be adopted in the mid‐term. High risk: Control measures should be adopted in the short term or immediately.

^c^ Uncertainty zone: Control measures classified in Zone B of the RBA cost–benefit matrix require intensive analysis to obtain more information and reduce uncertainties before making any decision. Due to the scarcity of additional information or a high level of uncertainty, precautionary measures should be considered.

*Source*: Adapted from Tondo and Gonçalves (2021).

Finally, the analyses in the present study demonstrated that the RBA and AHP approaches achieved the same results regarding the classification of the most important criteria for choosing the best *rapid Salmonella* analysis method. However, according to the experts’ opinions, the RBA method was easier and faster to perform, whereas the AHP method allowed for the quantification of the results. The AHP was considered more challenging due to its pairwise comparison of criteria. The number of judgments required is equal to (*n* ^2^ − *n*)/2 for an *n* × *n* matrix, where *n* is the number of rows and columns (Saaty 1987, 1990). The challenge at this stage is to maintain consistency in judgment. The greater the number of elements to be compared, the more likely the pairwise evaluation becomes inconsistent, requiring judgment reviews to ensure a consistency rate lower than 10% (CR < 0.1) (Saaty 1987, 1990, 2013). In the present study, this resulted in 21 pairwise comparisons for each evaluator. Therefore, AHP is usually applied using a support system to facilitate pairwise evaluations. In the current study, the AHPWEB software was used (2022‐04‐20: Alpha version) (Françozo et al. 2023). Furthermore, the AHP remains subject to the imprecision of human judgment, and different experts may therefore evaluate the criteria differently.

It is important to note that both the RBA and the AHP methodologies are decision‐support tools. Thus, in solving food safety problems, where managers need to make choices in face of available conditions and alternatives, these tools may assist the decision‐making process through a well‐structured method, and the outputs can be used to support the decision adopted.

## 4 Conclusion

According to the results, the adoption of rapid *Salmonella* detection methods by large‐scale poultry companies is advantageous, bringing high to very high benefits and low to high costs. Considering these results, RBA recommended the implementation and use of rapid methods in these companies. In the first analysis, the RBA was unable to identify benefits in changing one well‐known method for another one, because the well‐known methods present similar characteristics and advantages to the companies. Additional information required by the RBA and provided by AHP yielded similar results, indicating that the decision to implement a new method should be based on the best business proposals. However, the study also demonstrated that a faster method would bring high to very high benefits and could be the companies’ choice, even costing more than others, if its characteristics include adequate supply capacity, international validations, equipment on loan, and adequate technical support. That is, the benefits of the new faster method outweighed the risks of changing a well‐known method. Finally, the AHP confirmed the robustness of the RBA results in the study, with the latter being easier to perform, which can be useful in the decision‐making process during the risk management of FSI.

## Funding

This research did not receive specific grants from public, commercial, or not‐for‐profit funding agencies.

## Conflicts of Interest

The authors declare no conflicts of interest.

## Supporting information




**Supporting file**: risa70222‐supp‐0001‐SuppMat.docx

**Supporting file**: risa70222‐supp‐0002‐SuppMat.docx

**Supporting file**: risa70222‐supp‐0003‐SuppMat.xlsx

## Data Availability

The data that support the findings of this study are available in the supplementary material of this article, and additional information can be made available by the corresponding author upon reasonable request.
