# Supplementary material for: Using Risk–Benefit Analysis and the Analytical Hierarchy Process to Decide on the Implementation of Rapid Salmonella Detection Methods in Large Poultry Industries
Source: Risk Anal. 2026 Mar 24;46(4):e70222. doi: 10.1111/risa.70222 (PMC13010784; doi:10.1111/risa.70222)
Supplement: Supplementary file 2 — Supporting file: risa70222‐supp‐0002‐SuppMat.docx [file RISA-46-0-s001.docx]

**Classification of Criteria for Rapid Tests for *Salmonella* spp.**

**INFORMED CONSENT FORM – ICF**

BASED ON THE GUIDELINES CONTAINED IN RESOLUTION CNS Nº466/2012, MS.

Dear participant:

A research study is currently underway entitled *“Using Risk-Benefit Analysis (RBA) and the Analytical Hierarchy Process (AHP) to decide on the implementation of rapid* Salmonella *detection methods in large poultry industries.”*

In this questionnaire, you will classify the size of your company or the company where you work, and you will be invited to express your preference regarding evaluation criteria for a control measure addressing a food safety hazard. For each criterion, you will choose the option that best reflects your evaluation, classifying it as: Irrelevant, Moderately Important, Important, Very Important or Essential. The criteria to be analyzed for the selection of the best rapid test are:

- Supply Capacity
- International Validation
- Cost per Sample
- Time to Obtain the Result
- Ease of Performing the Test
- Availability of Technical Support
- Cost of the Equipment Used in the Test

The estimated time to complete the questionnaire is approximately 10 minutes.

Your participation will occur through a Google Forms link. Only the researcher will have access to the information provided through the link. We emphasize that your email will not be shared and will be used solely for accessing the electronic form.

No response will be individually identified in the dissemination of scientific data. Please note that your participation in the study is voluntary; therefore, you are not required to provide information and/or collaborate with the activities requested by the researcher. If you decide not to participate in the study, or withdraw at any time, you will not suffer any harm.

Although there are no direct benefits to participating, you will be indirectly contributing to scientific production. By responding to the survey, you will not receive any financial or other benefit and will not be exposed to any risk, as no personal identification data will be requested. The researcher is committed to disseminating the research results in a format accessible to the group or population studied.

Although there are no direct benefits from participating, indirectly you will be contributing to scientific knowledge production. By answering the questionnaire, you will not receive financial or any other type of benefit, nor will you be exposed to any risk, given the absence of requests for personal identification data. The researcher commits to disseminating the research results in a format accessible to the group or population studied.

The project was evaluated by an institutional Research Ethics Committee, a collegiate body with consultative, deliberative, and educational functions, whose purpose is to evaluate, issue opinions on, and monitor research projects involving human subjects with regard to their ethical and methodological aspects, conducted within the institution.

Sincerely,
The Researchers

Do you agree to participate:

I Agree I Do Not Agree

**Classification of Criteria for Rapid Tests for *Salmonella* spp.**

This questionnaire aims to classify the criteria that should be considered in the evaluation of rapid tests for *Salmonella* spp.

1. **What is the size of your company?**

- Microenterprise (up to 19 employees)
- Small enterprise (20 to 99 employees)
- Medium enterprise (100 to 499 employees)
- Large enterprise (500 employees or more)

1. **Mark the option that best reflects your evaluation for each of the criteria below:**

**Supply Capacity**

- Irrelevant
- Moderately Important
- Important
- Very Important
- Essential

**International Validation**

- Irrelevant
- Moderately Important
- Important
- Very Important
- Essential

**Cost per Sample**

- Irrelevant
- Moderately Important
- Important
- Very Important
- Essential

**Time to Obtain the Result for *Salmonella* spp.**

- Irrelevant
- Moderately Important
- Important
- Very Important
- Essential

**Ease of Performing the Test**

- Irrelevant
- Moderately Important
- Important
- Very Important
- Essential

**Availability of Technical Support**

- Irrelevant
- Moderately Important
- Important
- Very Important
- Essential

**Cost of the Equipment Used in the Test**

- Irrelevant
- Moderately Important
- Important
- Very Important
- Essential

1. **Do you wish to indicate another criterion for evaluation?**

- Yes
- No

**If you marked Yes, please indicate the new criterion: ______________**

1. **Please mark the option that best represents the criterion you indicated. This criterion is:**

- Irrelevant
- Moderately Important
- Important
- Very Important
- Essential
